# Measurement of Bradykinin Formation and Degradation in Blood Plasma: Relevance for Acquired Angioedema Associated With Angiotensin Converting Enzyme Inhibition and for Hereditary Angioedema Due to Factor XII or Plasminogen Gene Variants

**DOI:** 10.3389/fmed.2020.00358

**Published:** 2020-07-17

**Authors:** François Marceau, Georges E. Rivard, Julie M. Gauthier, Karen E. Binkley, Arnaud Bonnefoy, Isabelle Boccon-Gibod, Laurence Bouillet, Matthieu Picard, Ghislain Levesque, Hannah Laure Elfassy, Hélène Bachelard, Jacques Hébert, Konrad Bork

**Affiliations:** ^1^Axe Microbiologie-Infectiologie et Immunologie, CHU de Québec-Université Laval, Québec, QC, Canada; ^2^CHU Sainte-Justine, Université de Montréal, Montréal, QC, Canada; ^3^Molecular Diagnostic Laboratory, Division of Medical Genetics, Department of Pediatrics, Sainte-Justine University Hospital Center, University of Montreal, Montreal, QC, Canada; ^4^Division of Clinical Immunology and Allergy, Department of Medicine, St. Michael's Hospital, University of Toronto, Toronto, ON, Canada; ^5^National Reference Center for Angioedema (CREAK), Grenoble University Hospital, Grenoble, France; ^6^Service d'Immunologie Clinique etl allergie, Département de Médecine, Hôpital Maisonneuve-Rosemont, Montréal, QC, Canada; ^7^CLSC Sainte-Rose, Laval, QC, Canada; ^8^Département d'Immunologie-Allergie, Hôpital du Sacré-Coeur de Montréal, Montréal, QC, Canada; ^9^Axe Endocrinologie et Néphrologie, CHU de Québec-Université Laval, Québec, QC, Canada; ^10^Service d'Allergie, CHU de Québec-Université Laval, Québec, QC, Canada; ^11^Department of Dermatology, University Medical Center, Johannes Gutenberg University, Mainz, Germany

**Keywords:** B_2_ receptors, bradykinin, hereditary angioedema with normal C1 inhibitor level, kallikreins, tissue plasminogen activator, plasmin

## Abstract

Bradykinin (BK)-mediated angioedema (AE) states are rare acquired or hereditary conditions involving localized edema of the subcutaneous and submucosal tissues. Citrated plasma from healthy volunteers or patients with hereditary angioedema (HAE) with normal level of C1-inhibitor (C1-INH) was used to investigate pathways of BK formation and breakdown relevant to AE physiopathology. The half-life of BK (100 nM) added to normal plasma was 34 s, a value that was increased ~12-fold when the angiotensin converting enzyme (ACE) inhibitor enalaprilat (130 nM) was added (enzyme immunoassay measurements). The BK half-life was similarly increased ~5-fold following 2 daily oral doses of enalapril maleate in healthy volunteers, finding of possible relevance for the most common form of drug-associated AE. We also addressed the kinetics of immunoreactive BK (iBK) formation and decline, spontaneous or under three standardized stimuli: tissue kallikrein (KLK-1), the particulate material Kontact-APTT™ and tissue plasminogen activator (tPA). Relative to controls, iBK production was rapid (10–20 min) and very intense in response to tPA in plasma of female heterozygotes for variants in gene *F12* coding for factor XII (FXII) (p.Thr328Lys, 9 patients; p.Thr328Arg, one). An increased response to Kontact-APTT™ and an early tPA-induced cleavage of anomalous FXII (immunoblots) were also observed. Biotechnological inhibitors showed that the early response to tPA was dependent on plasmin, FXIIa and plasma kallikrein. Results from post-menopausal and pre-menopausal women with HAE-FXII were indistinguishable. The iBK production profiles in seven patients with the plasminogen p.Lys330Glu variant (HAE-PLG) did not significantly differ from those of controls, except for an unexpected, rapid and lanadelumab-resistant potentiation of KLK-1 effect. This enzyme did not cleave plasminogen or factor XII, suggesting a possible idiosyncratic interaction of the plasminogen pathogenic variant with KLK-1 activity. KLK-1 abounds in salivary glands and human saliva, hypothetically correlating with the clinical presentation of HAE-PLG that includes the swelling of the tongue, lips and contiguous throat tissues. Samples from HAE patients with normal C1-INH levels and *F12* gene did not produce excessive iBK in response to stimuli. The *ex vivo* approach provides physiopathological insight into AE states and supports the heterogeneous physiopathology of HAE with normal C1-INH.

## Introduction

**Figure 1:**
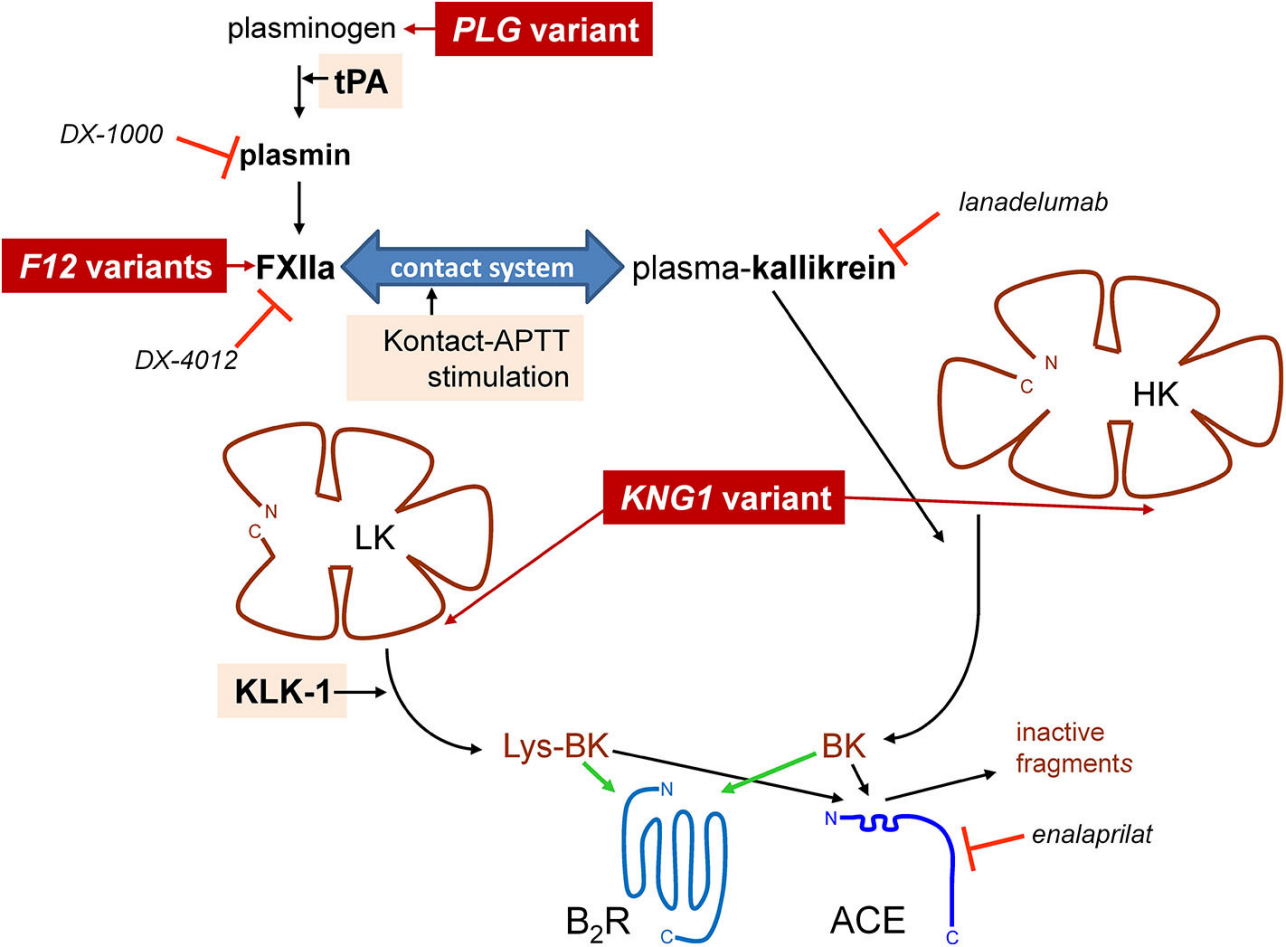
Schematic representation of established pathways of formation of BK-related peptides with indication of some of the known proteins for which pathogenic variants cause HAE-nC1-INH (dark red background) and of the stimuli applied in this work to trigger kinin formation (pale shaded background). Selected biotechnological protease inhibitors are also represented.

Bradykinin (BK)-mediated angioedema (AE) is a rare acquired or hereditary condition involving localized edema of the subcutaneous and submucosal tissues. The most common cause of acquired AE is drug-induced, in the context of treatment of patients with angiotensin-I converting enzyme (ACE) inhibitors for cardiovascular conditions (1). ACE-mediated cleavage is by far the most effective clearance pathways for kinin peptides in blood plasma and *in vivo*, BK being a high affinity substrate for this carboxydipeptidase (2, 3). Hereditary angioedema (HAE) is a rare autosomal dominant disorder most often caused by variants of the *SERPING1* gene encoding C1-inhibitor (C1-INH) with impaired production (type I HAE-C1-INH) or dysfunctional (type II HAE-C1-INH) forms of this serpin, a polyvalent inhibitor of proteases of the contact system, fibrinolysis and complement. The haploinsufficiency of C1-INH in blood is causally associated with attacks that involve the stimulation of the endothelial BK B_2_ receptor (B_2_R) and ensuing increased microvascular permeability. Even rarer forms of HAE with normal C1-INH (HAE-nC1-INH) are caused by mutation of genes encoding coagulation factor XII (*F12*) (4), plasminogen (*PLG*) (5), or of kininogens (*KNG1*) (6), presumably facilitating BK production (Figure 1). Indeed, some variants of factor XII (FXII) that are associated with HAE-nC1-INH introduce new sites of cleavage by plasmin that accelerate cleavage by this protease and also perhaps by thrombin, a plausible basis for a gain of function (7–9). The *PLG* K330E variant is associated with a particular form of HAE that mainly affects the tongue, lips and larynx (5, 10–12). A second class of causal genes is suggested by the angiopoietin 1 gene (*ANGPT1*) mutation associated with a very rare form of HAE-nC1-INH: the structural basis of the endothelial permeability barrier may rather be affected in this case (13).

While the physiopathology of HAE has been investigated by measuring the activity of plasma kallikrein (14, 15) and the consumption of high molecular weight kininogen (HK) in patients' blood (15–17), the measurement of BK is more rarely assessed, either under *in vitro* stimulation or after addition of synthetic kinins (2, 7, 18, 19). It offers a different point of view because this incriminated vasoactive mediator is short-lived and will accumulate only if its formation rate exceeds that of its breakdown. No significant spontaneous release of kinin was previously seen in whole blood or plasma sampled during remission from HAE-C1-INH patients upon incubation at 37°C but *in vitro* activation of fibrinolysis induced in these patients an earlier and brisker production of BK than that seen in normal controls (20, 21). The formation of immunoreactive BK (that includes Lys-BK) induced by contact system activation or by recombinant tissue kallikrein (KLK-1) did not differ between the 2 groups. This line of evidence combines with others to support the hypothesis that plasmin has an active role in the pathogenesis of HAE-C1-INH attacks (22). In the present work, we quantified the half-life (t_1/2_) of BK in plasma samples as influenced by an ACE inhibitor added *in vitro* or administered *in vivo* to show the transient nature of BK accumulation; this has also relevance to the physiopathology of ACE inhibitor-induced AE. Further, in the presence of all endogenous protease inhibitors of plasma, we also addressed the kinetics of BK formation under three standardized forms of stimulation in the plasma of HAE-nC1-INH due to *F12* or *PLG* variants in patient samples large enough for statistical analysis. Additional HAE-nC1-INH patients with unknown causal gene(s) were also studied to better address the diversity in the physiopathology of this disorder.

## Methods

### Human Subjects

The local ethical review board (Comité d'éthique de la recherche, CHU de Québec-Université Laval) granted ethical approval to carry out the study involving blood donations from adult healthy volunteers and HAE-nC1-INH patients 16 years old or older from Canada (file no. 2020-4696). Local ethical approval applied to plasma donors from abroad, with a material transfer agreement. All subjects gave written informed consent. The control group used for the kinetics of immunoreactive bradykinin (iBK) formation was entirely novel in regard to those of previous studies. Patient characteristics are listed in Table 1 and their blood was withdrawn when in remission. HAE patients who received a prophylactic treatment of plasma-derived C1-INH (Berinert, CLS Behring, Ottawa, Canada) continued to do so during the study; however, tranexamic acid was interrupted at least 48 h before blood sampling (Table 1). Female patients heterozygotes for p.Thr328Lys in FXII (also known as T309K in reference to mature FXII sequence; 1032C>A in *F12*) were recruited, with one additional subject harboring the p.Thr328Arg substitution. Patients 1 and 2 with HAE-FXII (p.Thr328Lys mutation) were previously described as patients II:7 and II:8, respectively (23, 24). All HAE-FXII female patients suffered from an essentially estrogen-dependent form of the disease, during pregnancies, oral contraception or estrogen administration for menopausal symptoms (23). None of these conditions were present at the time of blood sampling. In addition, the female patient 8 (p.Thr328Lys, Table 1) has never been symptomatic but was identified as the mother of affected daughters. The heterozygous p.Thr328Lys genotype of patient 3 was established by Fulgent Genetics (Temple City, CA). Patient 4 to 8 and 19, 20 were genotyped in the molecular diagnostic laboratory at CHU Sainte-Justine by bidirectional Sanger sequencing of the exon 9 of *F2* gene. The heterozygote genotype of patients with HAE-PLG was determined as described (5). Female patients 18 to 20, related over 3 generations, were clinically diagnosed with HAE-nC1-INH (normal C1-INH levels) and found to have a normal *F12* genotype (family d, Table 1), but the detection of rare causal variants of PLG, ANGPT1, and KNG1 was not performed. Mainly abdominal and oral cavity manifestations were observed in this family.

**Table 1:** Characteristics of patients with HAE-nC1-INH (females, except for 2 of the 7 HAE-PLG subjects).

**No**.	**Age range (years)**	**Genotype variant (all heterozygotes)**	**Approximate frequency of attacks**	**Prophylactic treatment**	C1-inh (U/ml)^*^
1^a^	66–70	*F12* T328K	None since menopause	None	0.93
2^a^	71–75	*F12* T328K	None since menopause	None	0.99
3	41–45	*F12* T328K	12 per year	Tranexamic acid (used on demand; not in the last 3 weeks)	0.96
4	36–40	*F12* T328K	Face and hand oedema during pregnancy; ~1/year outside of pregnancy/OC^**^	None	0.91
5^b^	26–30	*F12* T328K	Frequent face oedema while on OC. ~1/2.5 year while off OC	None	0.98
6^b^	36–40	*F12* T328K	Frequent face oedema while on OC; 2 episodes required endothacheal intubation; ~1/2.5 year while OFF OC	None	0.91
7^b^	31–35	*F12* T328K	4 episodes of face and tongue oedema during pregnancy; ~1/year while off pregnancy or OC	None	0.93
8^b^	51–55	*F12* T328K	Asymptomatic	N/A	0.95
9	36–40	*F12* T328K	Frequent face, tongue, hand, oedema while on OC or pregnant; ~1/year while off OC or pregnancy	None	0.95
10	31–35	*F12* T328R	Moderate severity	None	0.97
11	56–60	*PLG* K330E	Mild severity	None	0.99
12	46–50	*PLG* K330E	Mild severity	None	0.94
13	71–75	*PLG* K330E	Mild severity	None	0.99
14^c^	26–30	*PLG* K330E	Mild severity	None	0.90
15^c^	61–65	*PLG* K330E	Mild severity	None	0.75
16^c^	31–35	*PLG* K330E	Mild severity	None	0.70
17^c^	21–25	*PLG* K330E	Mild severity	None	0.72
18^d^	66–70	Unknown, C1-INH and *F12* normal	Now rare	None	0.94
19^d^	41–45	Unknown, C1-INH and *F12* normal	6 per year in spite of aggressive prophylaxis	Berinert (last dose: 24 h before sampling); tranexamic acid (stopped 48 h before sampling); icatibant several times/year	1.36
20^d^	16–20	Unknown, C1-INH and *F12* normal	2–3 per year in spite of aggressive prophylaxis	Berinert (last dose: 48 h before sampling); tranexamic acid (stopped 48 h before sampling); icatibant several times/year	1.24

* *normal range 0.70–1.30 U/ml*.

** *oral contraception*.

a−d *Individuals with the same superscript letter are related. All the others are unrelated between them*.

Biological evidence of C1-INH expression was obtained in the form of functional C1-INH measurements (based on a chromogenic substrate, Berichrom C1 Inhibitor, Siemens, Marburg, Germany).

### Enzyme Immunoassay (EIA) of BK

Venous blood was collected without contact with glass in plastic blood collection tubes with 0.11 M sodium citrate (BD Vacutainer Plus, BD Biosciences, Franklin Lake, NJ); the plasma was obtained by centrifugation (1,300 g, 20 min, room temperature), stored at −80°C until use and/or expedition on dry ice to Quebec City. The *in vitro* activation of plasma at 37°C (200 μl per experimental point sample) was performed precisely as described (21) in the presence of enalaprilat (final concentration 130 nM) to reduce the breakdown of BK. The samples were incubated for up to 120 min under rotary agitation (300 rpm) without any added stimulant or with recombinant human tissue kallikrein (KLK-1, 10 nM; gift from DiaMedica, Inc.), with the particulate contact system activator Pacific Hemostasis Kontact-APTT™ (2% v/v; used without the calcium supplement; composed mainly of magnesium aluminum silica and rabbit brain phospholipid; ThermoFisher Scientific) or with recombinant tissue plasminogen activator (tPA, 169 nM; Cathflo, Roche). The tPA concentration was reproduced from Molinaro et al. (19) and within the range reached in plasma during therapeutic fibrinolysis. Biotechnological inhibitors used in the mechanistic analysis of iBK formation were previously exploited (20, 21) and are listed in Table 2.

**Table 2:** Biotechnological inhibitors used in the present study.

**Inhibitor**	**Nature**	**Target**	**References**
Lanadelumab (DX-2930)	Human monoclonal antibody	Plasma kallikrein	(26)
DX-4012	Human monoclonal antibody	Factor XIIa	(27)
DX-2300	Human monoclonal antibody	Tissue kallikrein (KLK-1)	(28)
DX-1000	Kunitz-type inhibitor	Plasmin	(29)

An additional set of experiments aimed at measuring the rate of BK breakdown in the presence or absence of the ACE inhibitor enalaprilat or in plasma from selected human subjects: synthetic BK (100 nM) was added to 200 μl of plasma from healthy volunteers or HAE-nC1-INH patients and incubated at 37°C under agitation for 0–20 min.

In all experiments dealing with BK EIA, the reactions were stopped by adding 1 ml of cold ethanol, as described (21). After further incubation on ice-water for an additional period of at least 60 min, the plasma/ethanol mixtures were cleared of precipitated proteins by centrifugation (13,000 g, 1 min), evaporated to dryness (Speed Vac apparatus) and stored at −80°C until used for BK determination. Then, the samples were reconstituted with 200 μl of distilled water, further diluted 100-fold (1,000-fold for selected samples) with the supplied EIA buffer and directly applied in duplicate to the BK EIA as recommended by the manufacturer (Phoenix Pharmaceuticals, Burlingame, CA; cat. no. EK-009-01; 96-well plate format). The BK EIA is fully cross-reactive with Lys-BK, but not at all with des-Arg^9^-BK or Lys-des-Arg^9^-BK.

### Immunoblots for c-Fos Signaling

The BK EIA fully reacts with C-terminal BK fragments that have no biological activity (e.g., des-Arg^1^-BK) (20) whereas Lys-BK is approximately equipotent to BK at the human B_2_ receptor (25). Verifying the agonist status of representative extracts was based on the detection of c-Fos accumulation in cells that expressed the recombinant human B_2_ receptor (20) (methods, Supplementary Material).

### Immunoblots for Plasminogen and FXII

Samples of citrated plasma, optionally treated *in vitro* with one of the standardized stimuli, were directly loaded (0.5 μl of plasma mixed with lysis buffer) for 9% SDS-polyacrylamide gel electrophoresis (reducing conditions), transferred and the resulting membranes were washed as described in the Supplementary Material. Goat anti-human plasminogen antibodies, affinity purified, and peroxidase-conjugated, were added for overnight incubation in fresh blocking buffer (product GAPG-APHRP, Affinity Biologicals Inc., Ancaster, ON, Canada; dilution 1:5,000 or 1:10,000). The same samples were separately migrated and transferred for FXII immunoblotting using goat anti-human FXII, peroxidase conjugated antibodies (Affinity Biologicals, product GAFXII-HRP, dilution 1:10,000 or 1:20,000). The membranes were washed and the reaction with the antibodies revealed by chemoluminescence, as described in the Supplementary Material.

### Data Analysis

Numerical values are reported as means ± standard errors of the mean (S.E.M.). Sets of values were compared with Kruskall-Wallis test (non-parametric ANOVA) followed by Dunn's multiple comparison test to compare selected pairs of values. Pairs of values were compared using Mann-Whitney test (Prism 5.0, GraphPad Software Inc., San Diego, CA). The half-life (t_1/2_) of iBK was calculated as the first order decay procedure in a software suite (30).

## Results

### *In vitro* and *in vivo* Effect of an ACE Inhibitor on the Half-Life of BK Added to Plasma

**Figure 2:**
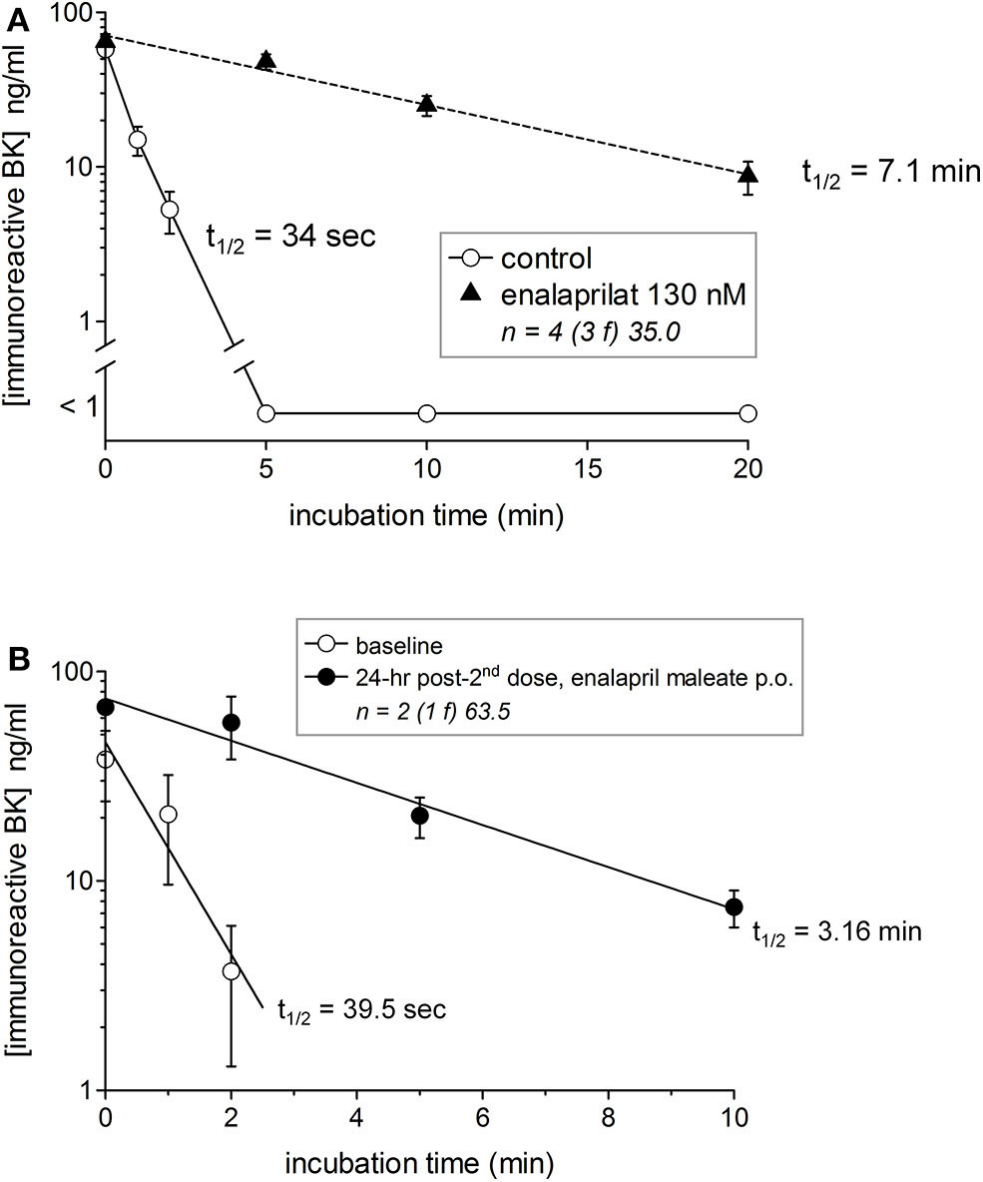
Degradation of synthetic BK (100 nM) added to human plasma from healthy subjects in the presence or absence of the ACE inhibitor enalaprilat **(A)** or to plasma from two human subjects before and 24 h after 2 oral doses of enalapril maleate (10 mg in 2 consecutive days) **(B)**. Samples were incubated at 37°C under agitation for the indicated time periods before extraction and EIA determination of BK. The number of replicated from different healthy donors is indicated by “n,” followed by the number of female donors between parentheses (f) and by the average age of donors (years). In the presence of the ACE inhibitor, the data are compatible with a first order decay with a half-life of 7.1 min, whereas BK was completely cleared in 5 min the absence of enalaprilat (t_1/2_ 34 s) **(A)**. A marked increase of BK t_1/2_ was also seen after oral enalapril dosing **(B)**.

We previously reported that iBK formation was negligible in either whole blood or plasma incubated at 37°C without stimulus both in healthy donors and HAE patients sampled during remission (HAE-C1-INH type I or II) (20, 21). This may be paradoxical because HK is spontaneously and progressively cleaved in the blood of patients with HAE-C1-INH (15, 16) or HAE-nC1-INH (17). A possible explanation is the efficient clearance of kinins. The breakdown of synthetic BK (100 nM) in the plasma of healthy volunteers was complete in <5 min with an estimated t_1/2_ of 34 s (Figure 2A). The t_1/2_ increased to 7.1 min when ACE was blocked *in vitro* with enalaprilat (Figure 2A), suggesting that iBK accumulation is the result of a production rate that surpasses the considerable buffer capacity of peptidases such as ACE. Two healthy volunteers took 2 daily doses of enalapril maleate (10 mg); they were normotensive but exhibited a slight reversible drop of their blood pressure during the experiment (Figure S1). BK t_1/2_ was 39.5 s in their plasma before dosing, but this value increased 4.8-fold 24 h after the last dose (Figure 2B), supporting the relevance of BK accumulation in the physiopathology of ACE inhibitor-induced AE.

### Kinetics of iBK Formation in the Stimulated Plasma of HAE-FXII and HAE-PLG Patients

**Figure 3:**
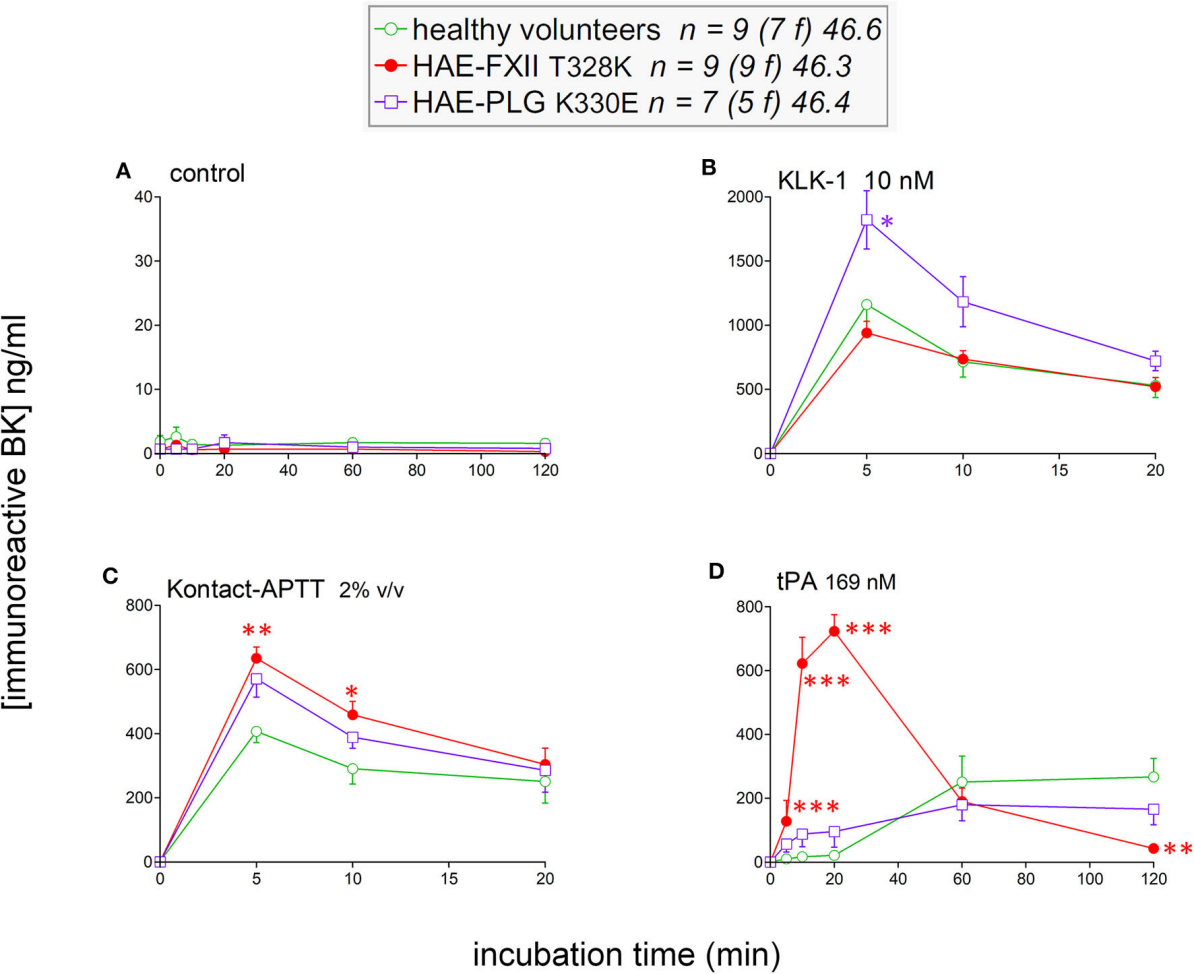
Kinetics of iBK concentrations as a function of time, stimulation, and diagnostic category in samples of plasma incubated at 37°C in the presence of enalaprilat (130 nM). Plasma samples originated from healthy subjects, HAE-FXII (T328K heterozygotes) or HAE-PLG (K330E heterozygotes) patients. *In vitro* stimulation conditions: **(A)** control; **(B)** KLK-1, 10 nM; **(C)** Kontact-APTT, 2% v/v; **(D)** tPA, 169 nM. For each experimental condition and time point, the Kruskall-Wallis test was applied to compare the effect of diagnostic category. When significant, Dunn's multiple comparison test was applied to compare the values from each type of HAE patients to those of the healthy controls. *P < 0.05; **P < 0.01; ***P < 0.001.

**Figure 4:**
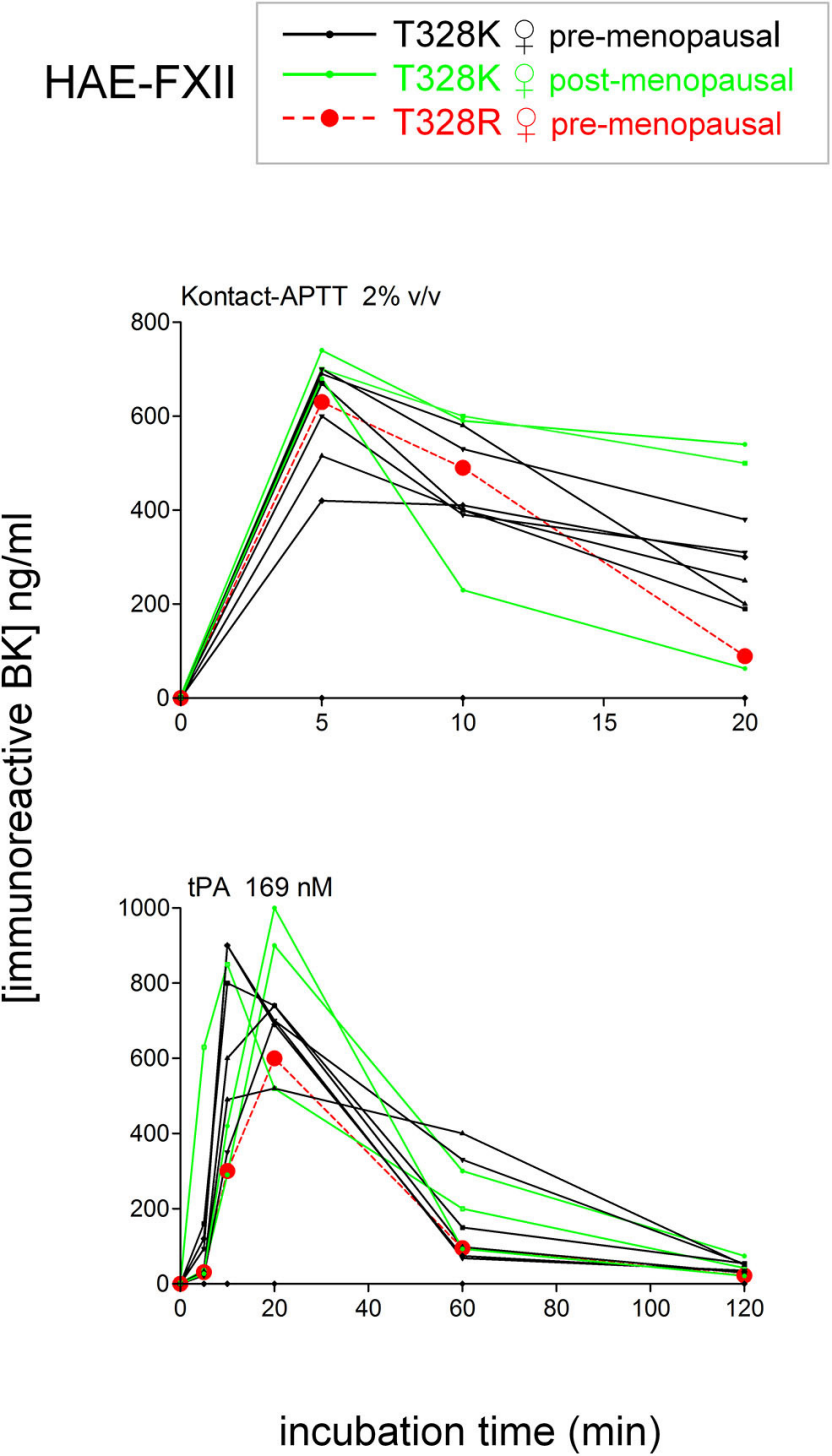
Effect of the T328R *F12* mutation (single female patient) or of the menopausal status in T328K heterozygotes on the stimulated production of iBK. Each curve is from an individual patient. Presentation as in Figure 3.

To isolate as much as possible the kinetics of iBK formation over its destruction, enalaprilat (130 nM) was present in plasma samples in the following experiments (except for immunoblot assays dealing with plasminogen and FXII cleavage). Plasma samples from both HAE-FXII patients heterozygous for the p.Thr328Lys variant and normal volunteers did not spontaneously release appreciable iBK in unstimulated plasma samples incubated for 0–2 h at 37°C (Figure 3A) but did so in a comparable fashion upon stimulation with KLK-1 (Figure 3B). Contact system activation with the standardized concentrations of the particulate material Kontact-APTT or tPA stimulation induced significant greater amount of iBK in HAE-FXII patients than in controls (Figures 3C,D, respectively). While plasmin is a known activator of normal FXII (31), the kinetics was particularly intense and rapid for tPA stimulation in FXII T328K heterozygotes (Figure 3D). Under the same experimental conditions, the plasma of HAE-C1-INH patients also released iBK faster than the controls in response to tPA, but the concentration did not exceed 300 ng/ml (21), whereas values in the HAE-FXII group were larger after 10 or 20 min of incubation (Figure 3D). The exhaustion of iBK formation later in the 2 h incubation period is specific to HAE-FXII plasma, suggesting the consumption of a factor critical for kinin production. The single individual with the T328R substitution was excluded from the results shown in Figure 3, but her plasma produced iBK in a manner qualitatively and quantitatively similar to patients with the T328K substitution (Figure 4). Examination of individual profiles of tPA-induced iBK production also indicated that 3 post-menopausal women with the T328K heterozygous substitution were indistinguishable from the pre-menopausal subjects with the same substitution (Figure 4). A bioassay involving the human recombinant B_2_ receptor expressed in HEK 293a cells, c-Fos accumulation in response to diluted reconstituted plasma extracts, confirmed that the very concentrated iBK in HAE-FXII plasma stimulated for short periods (5–20 min) with tPA, was highly biologically active, as compared to a lesser and protracted effect of extracts from incubated control plasma (Figure S2).

The iBK production profiles in seven patients with the plasminogen K330E variant (HAE-PLG) did not significantly differ from those of controls, except for an unexpected significant potentiation of KLK-1 effect at the shortest tested incubation period (5 min; Figure 3B).

### Effect of Biotechnological Inhibitors on Variant iBK Production in Plasma From HAE-FXII Patients

**Figure 5:**
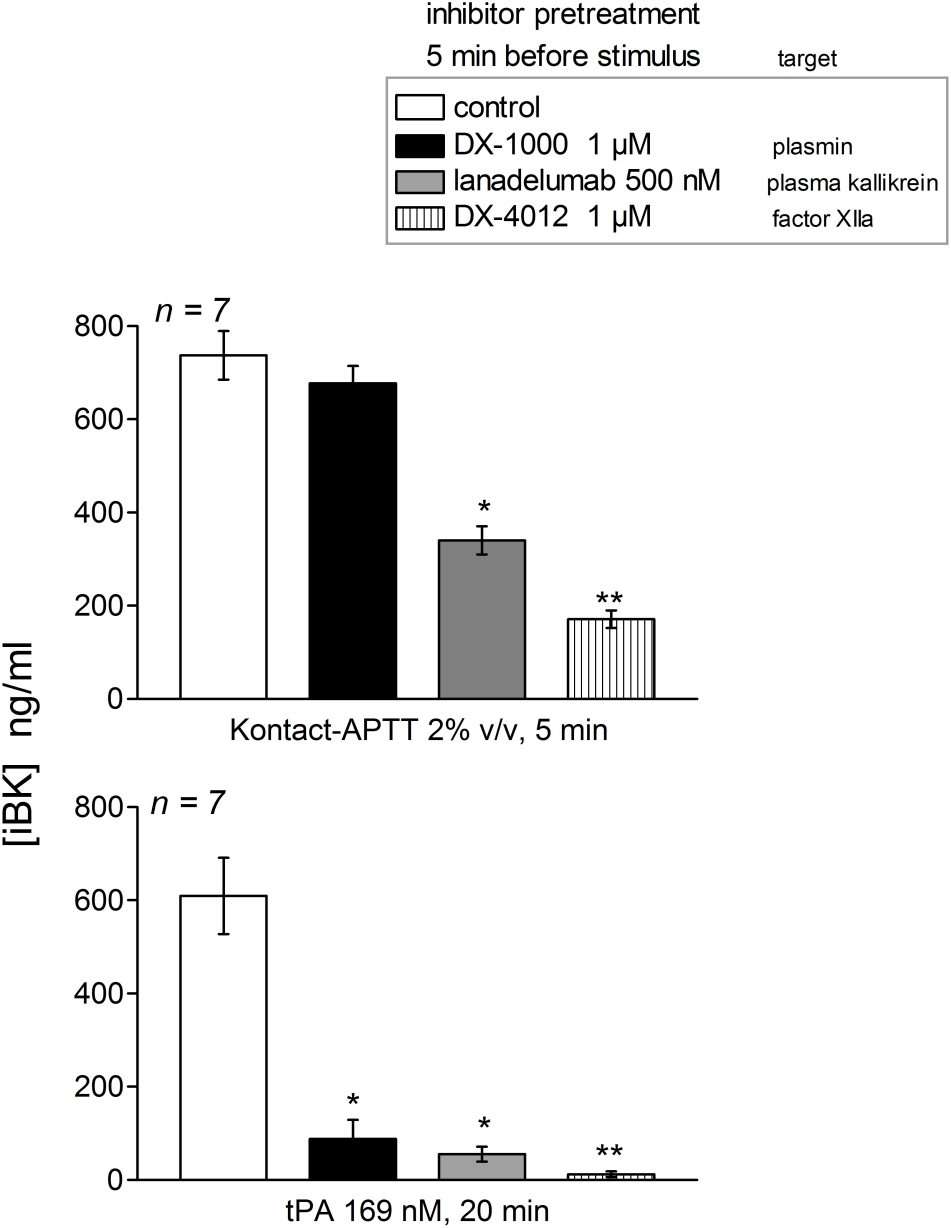
Effect of biotechnological inhibitors on maximal iBK generation induced by Kontact-APTT (top, 5 min incubation) or tPA (bottom, 20 min incubation) in plasma from patients with HAE-FXII (T328K). The inhibitors were added 5 min before the addition of stimuli to plasma at time zero. Enalaprilat (130 nM) was present in all tubes. Values are mean ± S.E.M. (number of subjects indicated by *n*). Kruskall-Wallis test applied to values from samples under each form of stimulation indicated significantly heterogeneous sets of values (*P* < 10^−4^ for Kontact-APPT, *P* < 0.001 for tPA). Dunn's multiple comparison test was applied to assess the effect of the inhibitors to their common control. *P < 0.05; **P < 0.001.

The early and high iBK release induced by tPA (20 min incubation) in plasma from patients with the T328K substitution was significantly decreased by the plasmin inhibitor DX-1000, the FXIIa inhibitor DX-4012 and lanadelumab (Figure 5). The early (5 min) response to Kontact-APTT was not abated by the plasmin inhibitor, but was significantly decreased by the 2 other inhibitors. This pattern of inhibition is qualitatively identical to that observed in the plasma or blood of healthy controls following a 60 min incubation period with tPA or a 5 min stimulation with Kontact-APTT (20, 21).

### Plasminogen and FXII Activation

**Figure 6:**
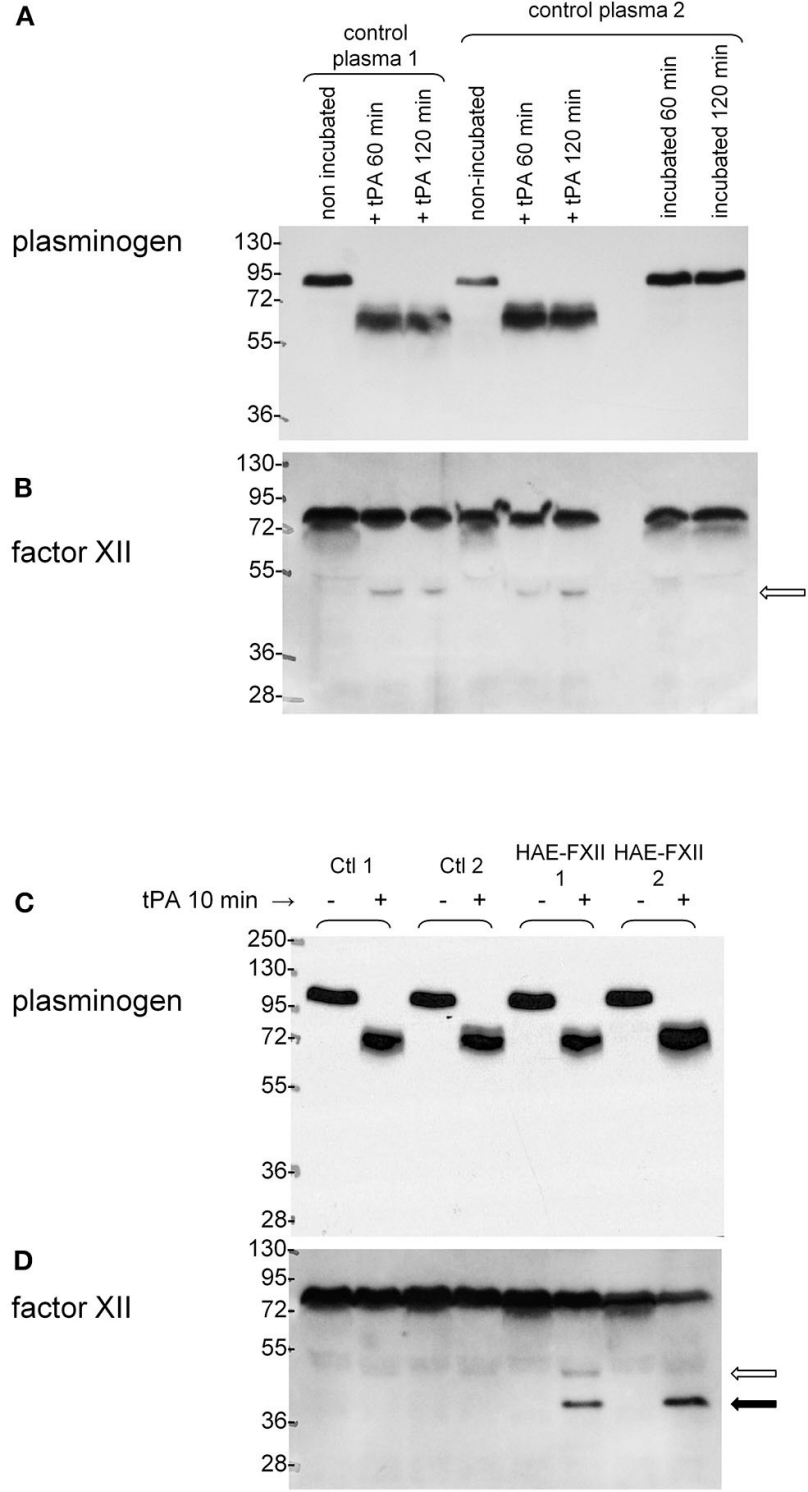
Parallel immunoblots of plasminogen and FXII in citrated plasma samples. 0.5 μl samples were loaded for migration in SDS-PAGE. **(A,B)** Samples from two healthy volunteers optionally treated *in vitro* with tPA (169 nM) with subsequent incubation for 60 or 120 min at 37°C under agitation. Other samples from volunteer 2 were incubated at 37°C without tPA to show stability at 37°C. **(C,D)** Plasminogen and FXII in the plasma of two different healthy volunteers and two patients with HAE-FXII (T328K) stimulated or not with tPA (169 nM) for 10 min. Representative of two separate experiments with different control and HAE-FXII plasma donors. Open arrow: heavy chain of cleaved normal FXII. Filled arrow: heavy chain of cleaved FXII pathogenic variant. See text for further analysis.

Plasminogen in the plasma of healthy individuals was revealed as a single 89–92 kDa band in immunoblots based on affinity-purified anti-plasminogen antibodies (Figure 6A). *In vitro* treatment with tPA (60 or 120 min) essentially cleaved all the endogenous plasminogen into bands found in the 58–69 kDa range, compatible with the heavy chains of the native protein or of the Lys-plasminogen metabolite generated by plasmin itself (32, 33). The antibodies did not react with the catalytically active light chain (26 kDa). This is evidence of maximal activation in a time period where tPA releases most iBK from control plasma (Figure 3D). Immunoblotting FXII in the same samples suggests that this protein is less stable than plasminogen in stored plasma, with minor degradation bands lighter than the 76 kDa FXII. However, incubation of control plasma at 37°C for 60–120 min generates some FXII light chain (52 kDa; Figure 6B). This reaction does not consume an important fraction of native FXII, possibly due to the presence of endogenous plasma inhibitors. Incubation with tPA (169 nM) cleaves (activates) all the plasminogen in control or HAE-FXII plasma samples in 10 min (Figure 6C), a short period during which little iBK is formed in healthy donor plasma (Figure 3D). This suggests that activation of FXII is the limiting step for kinin formation since iBK formation in tPA-stimulated control blood is dependent on FXIIa (20). Accordingly, little or none of the FXII heavy chain (52 kDa, open arrow) is seen in response to tPA in controls. In resting plasma from HAE-FXII patients, FXII is possibly a doublet with the slightly lower molecular form previously explained by the loss of the Thr^328^ O-glycosylation site (7); however, this is not well-resolved in the 9% gel (Figure 6D). The samples from heterozygous individuals (T328K) treated with tPA for 10 min evidenced the formation of a trace amount of the normal heavy chain and a more abundant lighter protein that may be identified as the heavy chain released from the FXII pathogenic variant (Figure 6D). The lower molecular weight form of native FXII corresponding to the variant form apparently disappeared in parallel (Figure 6D). The applied incubation duration of 10 min is associated with a massive iBK formation in these heterozygotes (Figure 3D). The anti-FXII antibodies did not react with the cleavage-induced light chain (28 kDa).

**Figure 7:**
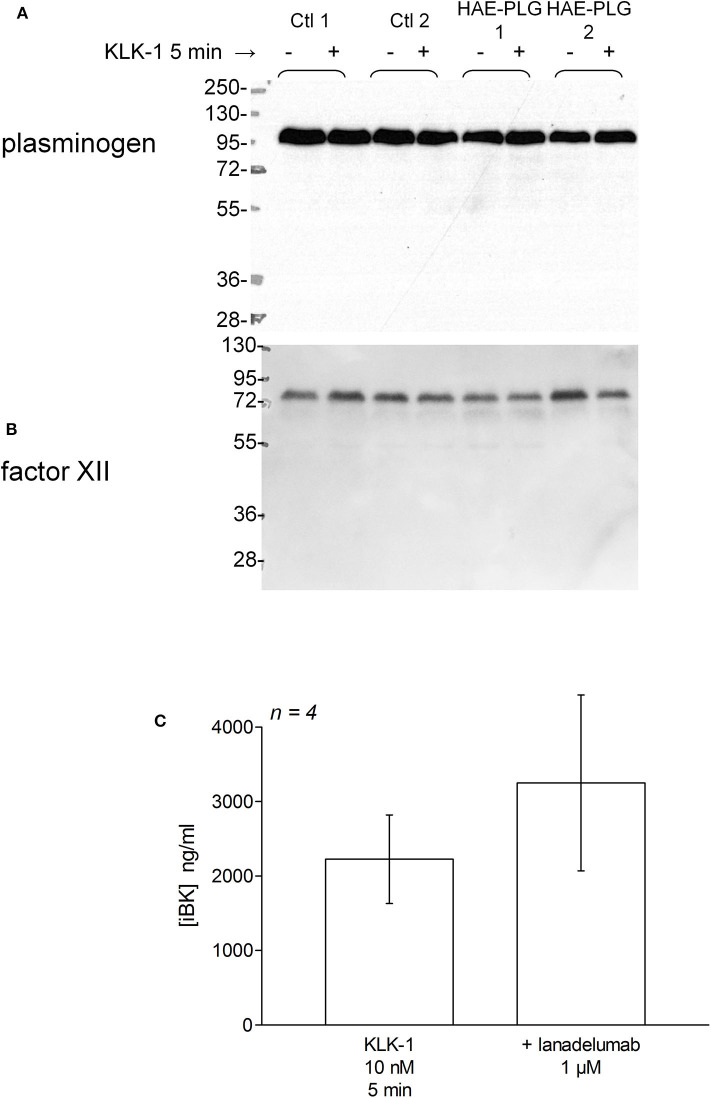
**(A,B)** Parallel immunoblots of plasminogen and FXII in citrated plasma samples from healthy volunteers or patients with HAE-PLG. Plasma samples were optionally treated with KLK-1 (10 nM) for 10 min before loading. **(C)** Lack of effect of lanadelumab on KLK-1-induced iBK formation during a 5 min incubation period. The inhibitor was added 5 min before the addition of KLK-1 to plasma at time zero. Presentation as in Figure 5. The values were not significantly different (Mann-Whitney test).

There was no evidence of plasminogen or FXII cleavage in plasma from patients with HAE-PLG, either spontaneously or under stimulation with KLK-1 (10 nM, 5 min) (Figures 7A,B). This stimulation period was sufficient for an increased production of iBK relative to controls in HAE-PLG patients (Figure 3B). The potentiated formation of iBK in response to KLK-1 (10 nM, 5 min) was not decreased by the treatment of HAE-PLG plasma with lanadelumab (Figure 7C), suggesting that the contact system is not recruited by the plasminogen pathogenic variant.

### Patients With HAE-nC1-INH of Unknown Genetic Cause

**Figure 8:**
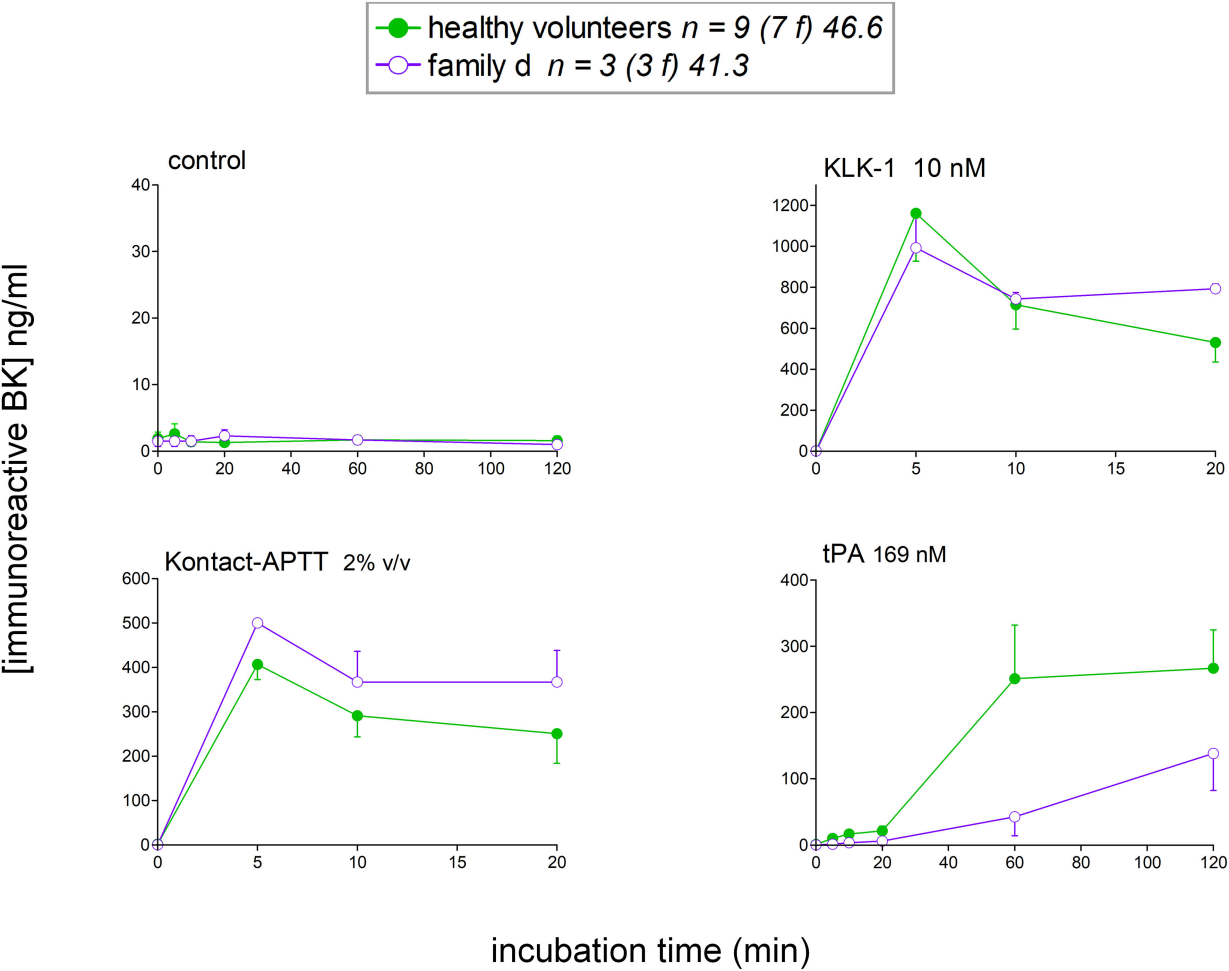
Kinetics of iBK concentrations in affected female members of family d with HAE-nC1-INH and tested negative for *F12* mutation. Presentation as in Figure 3. Family d is composed of three females over three generations (patients 18 to 20 in Table 1). The control group of healthy donors is repeated from Figure 3.

Female members from the family d (Table 1) with HAE-nC1-INH with unknown genetic cause (normal exon 9 in *F12*) were also included in the study (Figure 8). Assuming that the genetic basis is the same in this family, data from family members were pooled in this analysis. The profile of iBK release, either spontaneous or induced, did not exceed the values obtained from normal volunteers. On the contrary, tPA-induced iBK release was slower in this family, as opposed to what has been reported for HAE-C1-INH (20, 21), and HAE-FXII (Figure 3D). A metabolic anomaly leading to a decreased capacity to clear BK in these patients has not been documented as evidenced by the rate of degradation of synthetic BK (100 nM) in the absence of ACE inhibition (Figure S3). Also, the apparent delay in the generation of iBK in response to tPA was not explained by a large consumption of plasminogen or FXII, as revealed by immunoblots of plasma samples (Figure S4).

## Discussion

ACE blockade greatly increases BK persistence and pharmacological effect in human whole blood under a variety of *in vitro* stimulation conditions (20). Membrane-bound ACE produced by vascular endothelial cells is shed in an apparently regulated manner into blood plasma (34), allowing the use of blood/plasma/serum to monitor ACE activity in human subjects. Cyr et al. (2) found that the t_1/2_ of BK is 27 s and that this value is increased by 9-fold in the presence of enalaprilat; this is not dissimilar to our present results (33 s, 12-fold) considering several experimental differences (serum was then used instead of plasma and a higher BK concentration, 471 nM). There is additional evidence that the half-life of synthetic des-Arg^9^-BK is doubled when ACE is blocked in serum (2) and that the I/D polymorphism of the *ACE* gene modulates *ex vivo* BK t_1/2_ (18). We have extended the investigation to healthy volunteers who took two oral doses of enalapril maleate and verified the progressive and intense effect of ACE blockade on BK t_1/2_ over 48 h. These findings are possibly relevant for a role of vasodilator endogenous kinins in the therapeutic effects of ACE inhibitors in humans, as established by their reduced hypotensive effect when subjects are co-treated with the BK B_2_ receptor antagonist icatibant (35, 36). The prime importance of ACE in BK inactivation is in line with previous *in vitro* and *in vivo* findings (2, 3). The second most important kininase in live rats, aminopeptidase P, is only a distant third contributory inactivation pathway in human serum, possibly because this peptidase mostly remains membrane-bound (2, 3). The findings illustrate that BK is highly unstable and may contribute to the AAE-ACEI if, for some reason, the generation of kinins is triggered. Physical trauma, smoking, specific foods and co-administered drugs have been incriminated as triggering factors (37). The possible importance of the rapid clearance of BK for patients with HAE is the protection from the continuous production of BK, as judged from HK consumption (15–17).

The plasma of patients with HAE-nC1-INH was collected during a remission period for an *ex vivo* study of iBK formation as a function of three types of standardized stimulation; this was done in the presence of all endogenous protease inhibitors present in plasma and of an added ACE inhibitor. A limitation of the present study is that no genome wide sequencing has been applied to patient DNA, so the HAE subtype was diagnosed on the basis of the identification of known pathogenic variants of *F12* or *PLG*. Very rare combinations of pathogenic variants of different genes were not considered, although C1-INH levels were in the normal range in our HAE-nC1-INH sample (Table 1). As for HAE-FXII, our findings are largely compatible with and extend those of de Maat et al. (7): the p.Thr328Lys and p.Thr328Arg substitutions introduce in the FXII sequence a new cleavage site for plasmin, but not for plasma kallikrein, with a gain of function. We showed in whole plasma the rapid generation of a heavy chain of FXII-T328K that is lighter than the one generated from wild type FXII under tPA stimulation (immunoblots); this is due to the new cleavage site for plasmin that is N-terminally positioned relative to the classical site in either p.Thr328Lys or p.Thr328Arg variants (7). The consumption of normal FXII was not complete following tPA stimulation; this has been attributed to the effect of C1-INH (7). The apparent extensive consumption of the FXII pathogenic variant and more abundant variant FXII heavy chain production concurs with the proposition that the rate determining step for FXII activation is the plasmin cleavage at the mutated site. In our experiments, the massive formation of iBK under tPA stimulation (10–20 min) is attributable to plasmin, as shown by the inhibitory effect of the plasmin inhibitor DX-1000 on iBK release (Figure 5). The FXII inhibitor DX-4012 and lanadelumab, a plasma kallikrein inhibitor, suppressed as well the large iBK formation in the plasma of the HAE-FXII patients, showing that the latter is ultimately mediated by the classical contact system (Figure 1). In addition to exploiting the biotechnological inhibitors, our approach supported statistical analysis with a sufficient number of patients, and showed that the massive tPA-induced iBK formation mediated by plasmin is applicable as well to post-menopausal women, who have not experienced an HAE attack for decades (and never, in one case). Thus, the strict estrogen dependence of HAE attacks in the HAE-FXII (24) was not evidenced by the applied *ex vivo* analysis of plasma, and is possibly related to *in vivo* facilitated plasminogen activation. Estrogens reportedly increase fibrinolysis and factor XII during pregnancy, oral contraception or post-menopausal estrogen administration (38, 39). A significant decrease in fibrinogen levels has been noted during treatment with estrogen alone (40). The plausible triggering role of fibrinolysis in attacks of classical HAE due to C1-INH deficiency has also been recently discussed (20, 22). In addition, the presence of FXII-T328K increased significantly the early generation of iBK in response to the particulate material Kontact-APTT in a manner that was independent of plasmin, as judged by the lack of effect of DX-1000 (Figures 3, 5). This may indicate some facilitation of the contact system activity in the presence of the FXII pathogenic variants. Therapeutic fibrinolysis, sometimes associated with acquired AE or anaphylactoid side effects, has been previously modeled *in vitro*: adding tPA to plasma slowly released iBK, as in our control group, with some influence of aminopeptidase P expression on the intensity of the response, but not on its time course in individual volunteers (19). Whether variants of HAE causal genes predispose to side effects of therapeutic fibrinolysis is of potential interest.

In the plasminogen sequence, the residue 330 is not proximal to a physiological cleavage site or to the catalytic site: it is rather located within a potential adhesion site in the kringle 3 domain of the molecule (5). However, the K330E variant is associated with a distinct N-glysosylation pattern and an increased susceptibility to activation by urokinase-type plasminogen activator (uPA), but not by tPA (assessed only using a chromogenic substrate) (41). Accordingly, the effect of tPA on HAE-PLG plasma was not significantly different from that observed in control plasma (Figure 3D). If uPA activation of the K330E PLG variant translates into increased BK formation (not tested), a form of contact system activation may ensue via plasmin. In addition and unexpectedly, the early effect of recombinant tissue kallikrein (KLK-1) was enhanced in HAE-PLG. From the beginning of our investigations of HAE, we have kept monitoring the KLK-1 stimulation as an alternate pathway of kinin formation independent of the contact system and have not found profiles different from controls in HAE-C1-INH (20, 21). Lys-BK, formed by KLK-1 mainly from low molecular weight kininogen, fully cross reacts with the applied BK EIA and is a direct B_2_ receptor agonist (Figure 1). However, plasminogen or FXII are not cleaved by a KLK-1 concentration that releases an increased iBK output in 5 min (compare immunoblots with iBK kinetics, Figures 3B, 7A,B). Further lanadelumab did not inhibit KLK-1-induced iBK generation in plasma from HAE-PLG patients (Figure 7C), suggesting that the excess of kinin generation does not derive from secondary recruitment of the contact system. C1-INH administration to treat HAE-PLG attacks is not as effective as in HAE-C1-INH and some HAE-PLG patients are completely refractory (5, 42), which may support the alternate kinin formation pathway in some patients. If the increased KLK-1 kinin formation is confirmed in additional HAE-PLG patients and independent from unknown biases that may affect our sample, a molecular explanation involving the idiosyncratic interaction of the plasminogen pathogenic variant with KLK-1 itself or with its endogenous serpin inhibitor, kallistatin, must be investigated. In the meantime, it is worth noting that the salivary glands are ones of the most KLK-1 rich organs (https://www.proteinatlas.org/ENSG00000167748-KLK1/tissue) and that catalytically active KLK-1 is found in human saliva (43). The clinical presentation of HAE-PLG usually involves the tongues, lips and contiguous areas and a triggering role of salivary KLK-1 is plausible in this context and may be seen in the perspective of local manifestations of a systemic disease (44). The KLK-1 generated Lys-BK may be metabolized into the optimal agonist of the human B_1_ receptor, Lys-des-Arg^9^-BK (25); the expression of this inducible receptor may also be contributing, being possibly controlled by bacterial and inflammatory processes in the mouth. Previous *in vitro* work has shown that the contact system activation of whole human blood with Kontact-APTT does not produce an agonist for the human recombinant B_1_ receptor (c-Fos signaling) (20). This is consistent with the production of BK from HK by plasma kallikrein, and the fact that des-Arg^9^-BK, formed from BK by arginine carboxypeptidases, has only a low affinity for the human form of the B_1_ receptor (25).

In affected members of a family clinically diagnosed for HAE of unknown cause, the profile of iBK release, either spontaneous or induced, did not exceed the values obtained from normal volunteers. The functional C1-INH levels in these patients were within normal limits. This shows the limitation of the applied *ex vivo* approach where factors primarily affecting the endothelial permeability barrier cannot be evaluated. HAE-ANGPT1 is an example of possibly multiple HAE forms primarily affecting endothelium biology (45). Another example of the endothelial modulation of the risk of attacks in all BK-mediated AE states is the possible positive feed-back effect of BK on the number of binding sites for HK and LK (46) or the estrogen- and cytokine-induced facilitated activation of contact system via an action on endothelial cells (47). A better understanding of the physiopathology of the various HAE forms will support a more rational therapy and will justify the exhaustive genomic analysis of each affected patient/family in the future (45).

Our study based on the kinetics of iBK formation in plasma under standardized stimulation conditions revealed findings of possible physiopathological interest in HAE-FXII and HAE-PLG and supported the extreme heterogeneity of patients with HAE-nC1-INH. The half-life of synthetic BK in plasma is highly responsive to pharmacologic ACE inhibition *in vivo* and may be further investigated in AAE-ACEI and perhaps other drug-associated AE states (48).

## Data Availability Statement

The raw data supporting the conclusions of this article will be made available by the authors, without undue reservation.

## Ethics Statement

The studies involving human participants were reviewed and approved by Comité d'éthique de la recherche, CHU de Québec-Université Laval. Written informed consent to participate in this study was provided by the participants or, in one case, the participant's legal guardian/next of kin. Written informed consent was obtained from the individual(s) for the publication of any potentially identifiable images or data included in this article.

## Author Contributions

FM, HB, GR, IB-G, LB, and JH designed the experiments. FM, GR, HB, JG, and AB participated to the experimental work. GR, KEB, MP, GL, HE, JH, and KB recruited and characterized patients. FM analyzed results and wrote the manuscript draft. All authors read and approved the final manuscript.

## Conflict of Interest

FM served as a consultant for Pharvaris B.V. and received research funds from Shire/Takeda and Pharvaris B.V., outside the submitted work. GR has been a member of advisory boards (Baxalta, Bayer, Biogen Idec, CSL Berhing, Novo Nordisk, Octapharma, Pfizer) and received funding from Bayer, CSL Behring, and Pfizer (unrelated to the submitted work). KEB has been a consultant to, or speaker for Aralez, Shire/Takeda, CSL Behring, GSK, and Astra Zeneca. AB has been a member of advisory boards (Alexion Pharmaceuticals, Novo Nordisk), served as a consultant for Sanofi-Genzyme, received research funds from Alexion pharmaceuticals and has been a speaker/teacher for Diagnostica Stago. LB has been a member of advisory boards (Shire/Takeda, CSL Behring, Pharming, Novartis, GSK), receiving funding from Shire/Takeda, CSL Behring, Novartis, GSK, CHUGAI, Sanofi-Genzyme, and has been a speaker/teacher for Shire/Takeda, Novartis, GSK, Sanofi-Genzyme. MP received honoraria from Novartis, Pediapharm, and ALK for lectures unrelated to the submitted work. JH has been a speaker/teacher for CLS Behring, Novartis, and Shire; he has been a member of advisory committees (CLS Behring, Shire, and Novartis) and a clinical investigator for Merck (ALK), Stallergene, Boehringer-Ingelheim, GlaxoSmithKline (GSK), Novartis, Sanofi, AstraZeneca, CLS Behring, Shire, Roche, and Griffols (unrelated to the submitted work). KB has been a speaker for CSL Behring and Shire. The remaining authors declare that the research was conducted in the absence of any commercial or financial relationships that could be construed as a potential conflict of interest.

## References

[1] Bezalel S Mahlab-Guri K Asher I Werner B Sthoeger ZM. Angiotensin-converting enzyme inhibitor-induced angioedema. Am J Med. (2015) 128:120–5. 10.1016/j.amjmed.2014.07.011 25058867

[2] Cyr M Lepage Y Blais C Gervais N Cugno M Rouleau JL. Bradykinin and des-Arg^9^-bradykinin metabolic pathways and kinetics of activation of human plasma. Am J Physiol Heart Circul Physiol. (2001) 281:H275–83. 10.1152/ajpheart.2001.281.1.H275 11406494

[3] Fryer RM Segreti J Banfor PN Widomski DL Backes BJ Lin CW. Effect of bradykinin metabolism inhibitors on evoked hypotension in rats: rank efficacy of enzymes associated with bradykinin-mediated angioedema. Br J Pharmacol. (2008) 153:947–55. 10.1038/sj.bjp.0707641 18084312 PMC2267285

[4] Dewald G Bork K. Missense mutations in the coagulation factor XII (Hageman factor) gene in hereditary angioedema with normal C1 inhibitor. Biochem Biophys Res Commun. (2006) 343:1286–9. 10.1016/j.bbrc.2006.03.092 16638441

[5] Bork K Wulff K Steinmüller-Magin L Braenne I Staubach-Renz P Witzke G. Hereditary angioedema with a mutation in the plasminogen gene. Allergy. (2018) 73:442–50. 10.1111/all.13270 28795768

[6] Bork K Wulff K Rossmann H Steinmüller-Magin L Braenne I Witzke G. Hereditary angioedema cosegregating with a novel kininogen 1 gene mutation changing the N-terminal cleavage site of bradykinin. Allergy. (2019) 74:2479–81. 10.1111/all.13869 31087670

[7] de Maat S Björkqvist J Suffritti C Wiesenekker CP Nagtegaal W Koekman A. Plasmin is a natural trigger for bradykinin production in patients with hereditary angioedema with factor XII mutations. J Allergy Clin Immunol. (2016) 138:1414–23. 10.1016/j.jaci.2016.02.021 27130860

[8] Cichon S Martin L Hennies HC Müller F van Driessche K Karpushova A. Increased activity of coagulation factor XII (Hageman factor) causes hereditary angioedema type III. Am J Hum Genet. (2006) 79:1098–104. 10.1086/509899 17186468 PMC1698720

[9] Ivanov I Matafonov A Sun MF Mohammed BM Cheng Q Dickeson SK. A mechanism for hereditary angioedema with normal C1 inhibitor: an inhibitory regulatory role for the factor XII heavy chain. Blood. (2019) 133:1152–63. 10.1182/blood-2018-06-860270 30591525 PMC6405335

[10] Germenis AE Loules G Zamanakou M Psarros F González-Quevedo T Speletas M. On the pathogenicity of the plasminogen K330E mutation for hereditary angioedema. Allergy. (2018) 73:1751–3. 10.1111/all.13324 30009523

[11] Bodian DL Vilboux T Hauser NS. Genotype-first analysis of a generally healthy population cohort supports genetic testing for diagnosis of hereditary angioedema of unknown cause. Allergy Asthma Clin Immunol. (2019) 15:32. 10.1186/s13223-019-0346-1 31131012 PMC6524287

[12] Recke A Massalme EG Jappe U Steinmüller-Magin L Schmidt J Hellenbroich Y. Identification of the recently described plasminogen gene mutation p.Lys330Glu in a family from Northern Germany with hereditary angioedema. Clin Transl Allergy. (2019) 9:9. 10.1186/s13601-019-0247-x 30809376 PMC6374890

[13] Bafunno V Firinu D D'Apolito M Cordisco G Loffredo S Leccese A. Mutation of the angiopoietin-1 gene (ANGPT1) associates with a new type of hereditary angioedema. J Allergy Clin Immunol. (2018) 141:1009–17. 10.1016/j.jaci.2017.05.020 28601681

[14] Lara-Marquez ML Christiansen SC Riedl MA Herschbach J Zuraw BL. Threshold-stimulated kallikrein activity distinguishes bradykinin- from histamine-mediated angioedema. Clin Exp Allergy. (2018) 48:1429–38. 10.1111/cea.13219 29957871

[15] Joseph K Tuscano TB Kaplan AP. Studies of the mechanisms of bradykinin generation in hereditary angioedema plasma. Ann Allergy Asthma Immunol. (2008) 101:279–86. 10.1016/S1081-1206(10)60493-0 18814451

[16] Suffritti C Zanichelli A Maggioni L Bonanni E Cugno M Cicardi M. High-molecular-weight kininogen cleavage correlates with disease states in the bradykinin-mediated angioedema due to hereditary C1-inhibitor deficiency. Clin Exp Allergy. (2014) 44:1503–14. 10.1111/cea.12293 24552232

[17] Baroso R Sellier P Defendi F Charignon D Ghannam A Habib M. Kininogen cleavage assay: diagnostic assistance for kinin-mediated angioedema conditions. PLoS ONE. (2016) 11:e0163958. 10.1371/journal.pone.0163958 27685806 PMC5042432

[18] Brown NJ Blais C Jr Gandhi SK Adam A. ACE insertion/deletion genotype affects bradykinin metabolism. J Cardiovasc Pharmacol. (1998) 32:373–7. 10.1097/00005344-199809000-00006 9733349

[19] Molinaro G Gervais N Adam A Biochemical basis of angioedema associated with recombinant tissue plasminogen activator treatment: an *in vitro* experimental approach. Stroke. (2002) 33:1712–6. 10.1161/01.STR.0000017284.77838.87 12053016

[20] Charest-Morin X Hébert J Rivard GÉ Bonnefoy A Wagner É Marceau F. Comparing pathways of bradykinin formation in whole blood from healthy volunteers and patients with hereditary angioedema due to C1 inhibitor deficiency. Front Immunol. (2018) 9:2183. 10.3389/fimmu.2018.02183 30333824 PMC6176197

[21] Marceau F Bachelard H Rivard GÉ Hébert J. Increased fibrinolysis-induced bradykinin formation in hereditary angioedema confirmed using stored plasma and biotechnological inhibitors. BMC Res Notes. (2019) 12:291. 10.1186/s13104-019-4335-8 31133046 PMC6537381

[22] Maas C. Plasminflammation – an emerging pathway to bradykinin production. Front Immunol. (2019) 10:2046. 10.3389/fimmu.2019.02046 31507620 PMC6719523

[23] Binkley KE Davis AD. Clinical, biochemical, and genetic characterization of a novel estrogen-dependent inherited form of angioedema. J Allergy Clin Immunol. (2000) 106:546–50. 10.1067/mai.2000.108106 10984376

[24] Duan QL Binkley K Rouleau GA. Genetic analysis of Factor XII and bradykinin catabolic enzymes in a family with estrogen-dependent inherited angioedema. J Allergy Clin Immunol. (2009) 123:906–10. 10.1016/j.jaci.2008.12.010 19178938

[25] Leeb-Lundberg LM Marceau F Müller-Esterl W Pettibone DJ Zuraw BL. International Union of Pharmacology. XLV. Classification of the kinin receptor family: from molecular mechanisms to pathophysiological consequences. Pharmacol Rev. (2005) 57:27–77. 10.1124/pr.57.1.2 15734727

[26] Kenniston JA Faucette RR Martik D Comeau SR Lindberg AP Kopack KJ. Inhibition of plasma kallikrein by a highly specific active site blocking antibody. J Biol Chem. (2014) 289:23596–608. 10.1074/jbc.M114.569061 24970892 PMC4156074

[27] Kokoye Y Ivanov I Cheng Q Matafonov A Dickeson SK Mason S. A comparison of the effects of factor XII deficiency and prekallikrein deficiency on thrombus formation. Thromb Res. (2016) 140:118–24. 10.1016/j.thromres.2016.02.020 26950760 PMC4821716

[28] Sexton DJ Chen T Martik D Kuzmic P Kuang G Chen J. Specific inhibition of tissue kallikrein 1 with a human monoclonal antibody reveals a potential role in airway diseases. Biochem J. (2009) 422:383–92. 10.1042/BJ20090010 19527222

[29] Devy L Rabbani SA Stochl M Ruskowski M Mackie I Naa L. PEGylated DX-1000: pharmacokinetics and antineoplastic activity of a specific plasmin inhibitor. Neoplasia. (2007) 9:927–37. 10.1593/neo.07544 18030361 PMC2077884

[30] Tallarida RJ Murray RB Manual of Pharmacologic Calculations With Computer Programs, 2nd ed. New York, NY: Springer Verlag (1987).

[31] Kaplan AP Austen KF. A prealbumin activator of prekallikrein. II. Derivation of activators of prekallikrein from active Hageman factor by digestion with plasmin. J Exp Med. (1971) 133:696–712. 10.1084/jem.133.4.696 4251126 PMC2138966

[32] Violand BN Castellino FJ. Mechanism of the urokinase-catalyzed activation of human plasminogen. J Biol Chem. (1976) 251:3906–12. 132442

[33] Miles LA Castellino FJ Gong Y. Critical role for conversion of Glu-plasminogen to Lys-plasminogen for optimal stimulation of plasminogen activation on cell surfaces. Trends Cardiovasc Med. (2003) 13:21–30. 10.1016/S1050-1738(02)00190-1 12554097

[34] Ehlers MRW Gordon K Schwager SLU Sturrock ED. Shedding the load of hypertension: the proteolytic processing of angiotensin-converting enzyme. S Afr Med J. (2012) 102:461–4. 10.7196/SAMJ.5596 22668937

[35] Gainer JV Morrow JD Loveland A King DJ Brown NJ. Effect of bradykinin-receptor blockade on the response to angiotensin-converting-enzyme inhibitor in normotensive and hypertensive subjects. N Engl J Med. (1998) 339:1285–92. 10.1056/NEJM199810293391804 9791144

[36] Squire IB O'Kane KP Anderson N Reid JL. Bradykinin B2 receptor antagonism attenuates blood pressure response to acute angiotensin-converting enzyme inhibition in normal men. Hypertension. (2000) 36:132–36. 10.1161/01.HYP.36.1.132 10904025

[37] Hoover T Lippmann M Grouzmann E Marceau F Herscu P. Angiotensin converting enzyme inhibitor induced angio-oedema: a review of the pathophysiology and risk factors. Clin Exp Allergy. (2009) 40:50–61. 10.1111/j.1365-2222.2009.03323.x 19659669

[38] Gordon EM Ratnoff OD Saito H Donaldson VH Pensky J Jones PK. Rapid fibrinolysis, augmented Hageman factor (factor XII) titers, and decreased C1 esterase inhibitor titers in women taking oral contraceptives. J Lab Clin Med. (1980) 96:762–9. 7419960

[39] Jespersen J Kluft C. Increased euglobulin fibrinolytic potential in women on oral contraceptives low in estrogen levels of extrinsic and intrinsic plasminogen activators, prekallikrein, factor XII and C1 inactivator. Thromb Haemost. (1985) 54:454–9. 10.1055/s-0038-1657871 2417350

[40] Conard J Gompel A Pelisser C Mirabel C Basdevant A. Fibrinogen and plasminogen modifications during estradiol replacement therapy. Fertil Steril. (1997) 68:449–53. 10.1016/S0015-0282(97)00220-3 9314913

[41] Parsopoulou F Charignon D Tengo M Psarros F Maas C Gonzalez-Quevedo Drouet C. Plasminogen glycoforms alteration and activation susceptibility associated with the missense variant p.Lys330Glu in HAE-PLG patients. Allergy. (2020). 10.1111/all.14280. [Epub ahead of print]. 32181895

[42] Bork K Wulff K Witzke G Machnig T Hardt J. Treatment of patients with hereditary angioedema with the c.988A>G (p.Lys330Glu) variant in the plasminogen gene. Orphanet J Rare Dis. (2020) 15:52. 10.1186/s13023-020-1334-8 32066472 PMC7026952

[43] Pantano E Marchi S Biagini M Di Fede M Nardi Dei M Rossi Paccani S. NHBA is processed by kallikrein from human saliva. PLoS ONE. (2019) 14:e02003234. 10.1371/journal.pone.0203234 31369555 PMC6675046

[44] Hofman ZLM Relan A Zeerleder S Drouet C Zuraw B Hack CE. Angioedema attacks in patients with hereditary angioedema: local manifestations of a systemic activation process. J Allergy Clin Immunol. (2016) 138:359–66. 10.1016/j.jaci.2016.02.041 27246526

[45] Marcelino-Rodriguez I Callero A Mendoza-Alvarez A Perez-Rodriguez E Barrios-Recio J Garcia-Robaina JC. Bradykinin-mediated angioedema: an update of the genetic causes and the impact of genomics. Front Genet. (2019) 10:900. 10.3389/fgene.2019.00900 31611908 PMC6776636

[46] Zini JM Schmaier AH Cines DB. Bradykinin regulates the expression of kininogen binding sites on endothelial cells. Blood. (1993) 81:2936–46. 10.1182/blood.V81.11.2936.2936 8388750

[47] Joseph K Tholanikunnel BG Kaplan AP. Cytokine and estrogen stimulation of endothelial cells augments activation of the prekallikrein-high molecular weight kininogen complex: implications for hereditary angioedema. J Allergy Clin Immunol. (2017) 140:170–6. 10.1016/j.jaci.2016.09.032 27826093

[48] Bas M Greve J Strassen U Khosravani F Hoffmann TK Kojda G. Angioedema induced by cardiovascular drugs: new players join old friends. Allergy. (2015) 70:1196–200. 10.1111/all.12680 26119220

