# Supplementary material for: Measurement of Bradykinin Formation and Degradation in Blood Plasma: Relevance for Acquired Angioedema Associated With Angiotensin Converting Enzyme Inhibition and for Hereditary Angioedema Due to Factor XII or Plasminogen Gene Variants
Source: Front Med (Lausanne). 2020 Jul 17;7:358. doi: 10.3389/fmed.2020.00358 (PMC7380097; doi:10.3389/fmed.2020.00358)

Supplementary Material

Measurement of bradykinin formation and degradation in blood plasma: relevance for acquired angioedema associated with angiotensin converting enzyme inhibition and for hereditary angioedema due to factor XII or plasminogen gene variants

François Marceau, Georges-Étienne Rivard, Julie M. Gauthier, Karen Binkley, Arnaud Bonnefoy, Isabelle Boccon-Gibod, Laurence Bouillet, Matthieu Picard, Ghislain Levesque, Hannah Laure Elfassy, Hélène Bachelard, Jacques Hébert, Konrad Bork

*** Correspondence:** F. Marceau: francois.marceau@crchudequebec.ulaval.ca

Supplementary Methods: Immunoblots for c-Fos signaling

The construction of myc-tagged B_2 receptor expression vector containing the human receptor sequence has been recently reported, as well as the derivation of HEK293a cell lines that stably express the construction (Charest-Morin et al., 2015). The accumulation of the c-Fos transcription factor was measured in HEK 293a cells that stably expressed recombinant myc-tagged BK B_2 receptors following 1 hour of stimulation with plasma extracts or synthetic BK. For the analysis of c-Fos in total cell lysates, the culture medium was removed and adherent cells were washed once with ice-cold PBS. Then, boiling lysis buffer containing 10 mM Tris pH 7.4, 1.0 mM Na_3VO_4, 1 mM PMSF, one tablet of Complete Mini protease inhibitor cocktail per 10 ml (Roche Diagnostics, Mannheim, Germany) and 1.0% SDS was applied to adherent cells after the removal of PBS. The lysates were removed, transferred to Eppendorf tubes, homogenized by 5 passes through a 27-gauge needle, further incubated for 5 min at 100°C and then centrifuged at 12,000×g for 10 min. Total protein concentrations in supernatants were then determined using the bicinchoninic acid protein assay (Pierce, Rockford, IL, USA). The samples, standardized for protein concentration, were

denatured in 1% denaturation buffer (0.5% SDS, 1% v/v β-mercaptoethanol) at 95°C for 5 min. Twenty-five micrograms of total cellular proteins were run on a 9% SDS-polyacrylamide gel electrophoresis and transferred to a polyvinylidene difluoride membrane. The blots were then incubated 1 hour at room temperature in blocking buffer [washing buffer (10 mM Tris pH 7.5, 100 mM NaCl, 0.1% Tween 20) containing 5% skimmed milk]. The primary antibody was added for incubation overnight at 4°C in fresh blocking buffer. c-Fos expression was assessed using a rabbit monoclonal antibody (mAb) (clone 9F6, dilution 1:1,000, Cell Signaling Technology). The membranes were washed for 30 min in washing buffer at room temperature before adding the appropriate secondary antibody (horseradish peroxidase-conjugated, preadsorbed grade; Jackson ImmunoResearch Labs, West Grove, PA) for 1 hour at room temperature in blocking buffer. The membranes were washed in washing buffer for another 30 min and then the antibodies were revealed using the Western Blot Chemoluminescence Reagent Plus (NEN Life Science Products), as directed. Equal track loading was further verified by migrating and transferring the same samples separately and immunoblotting for β-actin (mAb from Sigma-Aldrich; dilution 1:50,000).

Supplementary Reference

Charest-Morin X, Raghavan A, Charles ML, Kolodka T, Bouthillier J, Jean M, et al. Pharmacological effects of recombinant human tissue kallikrein on bradykinin B2 receptors. *Pharmacol Res Perspect* (2015) 3:e00119. doi: 10.1002/prp2.119

Supplementary Results

Figure S1. Blood pressure measurements before, during and after oral dosing with enalapril maleate in two healthy volunteers subjected to the ex vivo measurement of BK degradation (Fig. 2B). Values are means ± s.e.m.

Fig. S1

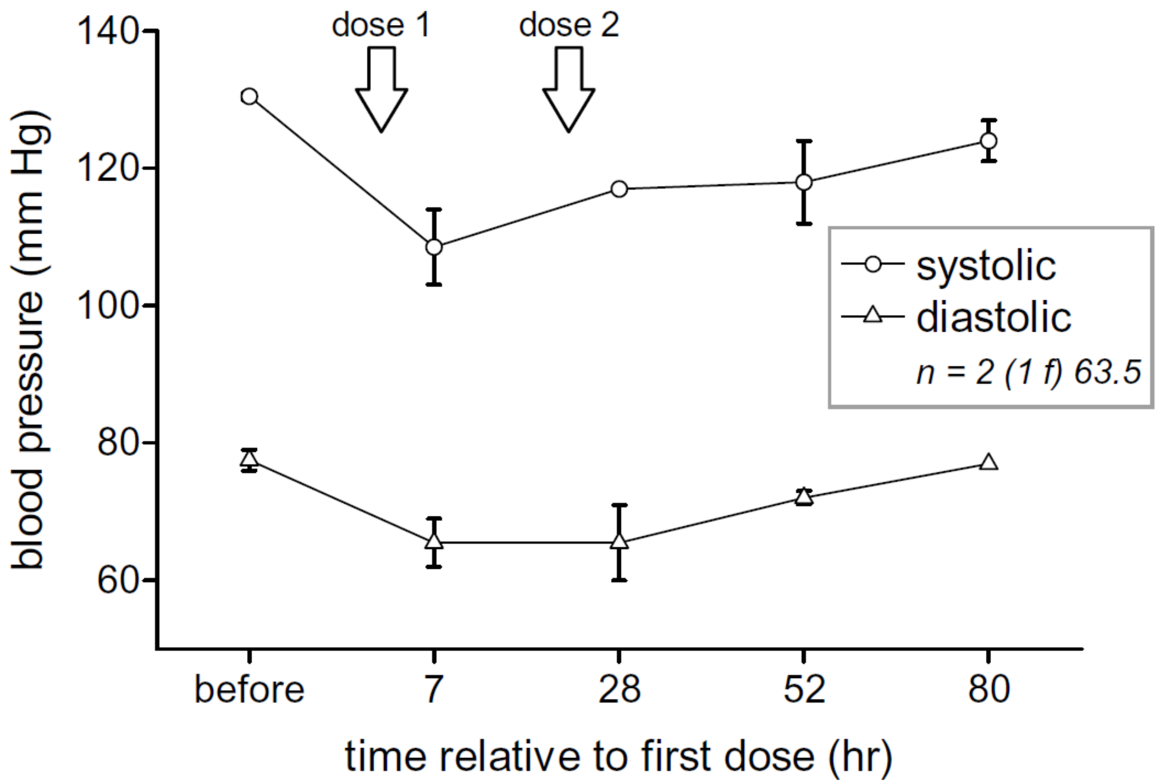

Figure S2. c-Fos accumulation in HEK 293a cells expressing recombinant myc-B_2Rs and stimulated for 60 min with diluted extracts (1:50) of plasma samples from a HAE-FXII patient (no. 2 in Table 1) or from a healthy subject. Plasma samples were incubated in the presence of tPA for the indicated time period before extraction (reconstituted plasma extracts processed as in Fig. 3).

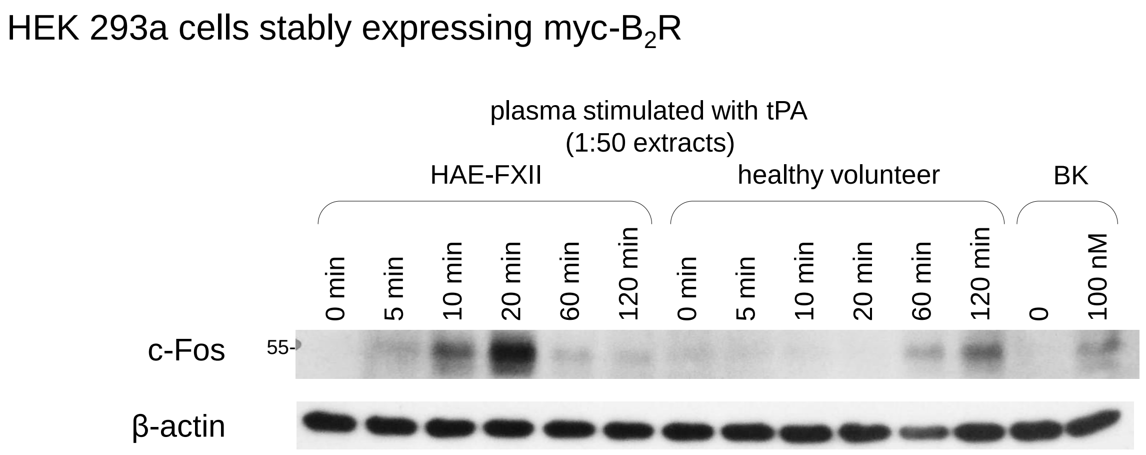

Figure S3. Degradation of synthetic BK (100 nM) in the absence of an ACE inhibitor in the plasma of the subjects representing a family with HAE-nC1-INH (family d in Table 1). Presentation as in Fig. 2.

Fig. S3

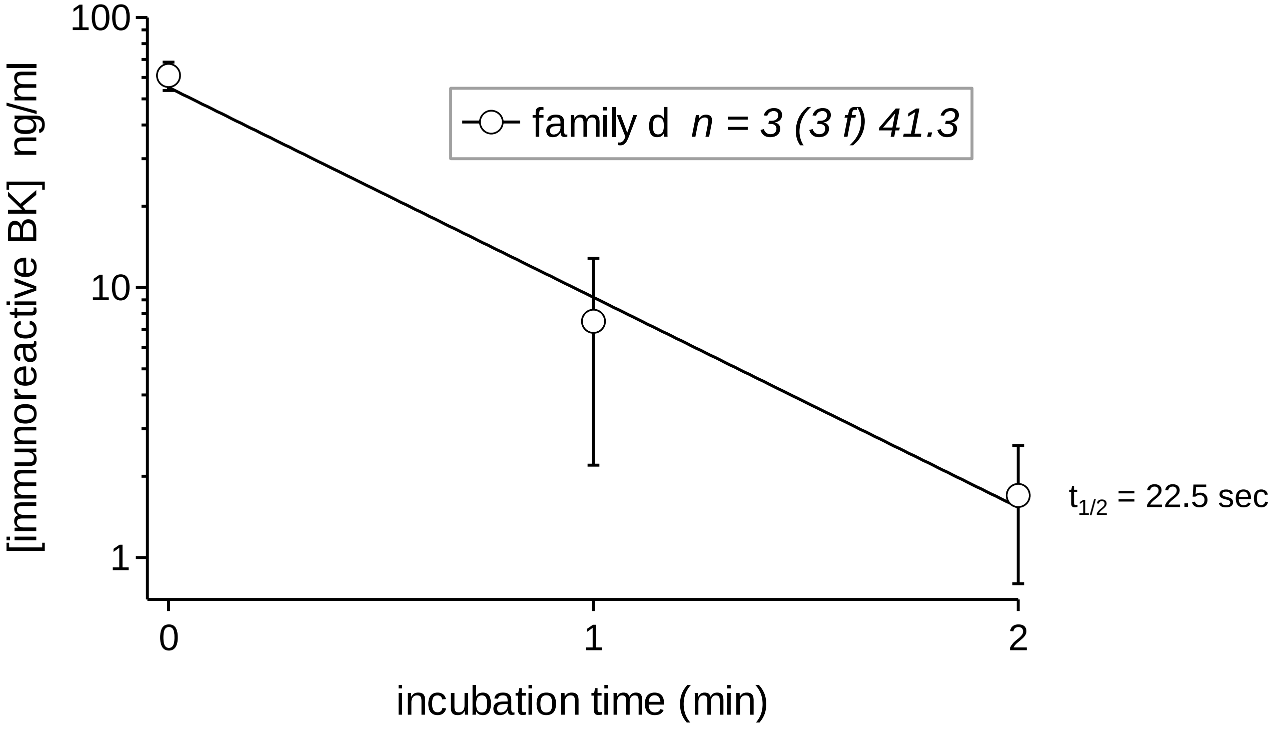

Figure S4. Lack of evidence for large consumption of plasminogen or FXII in controls and affected female members of the family d (Table 1) with HAE-nC1-INH tested negative for *F12* mutation.

Fig. S4

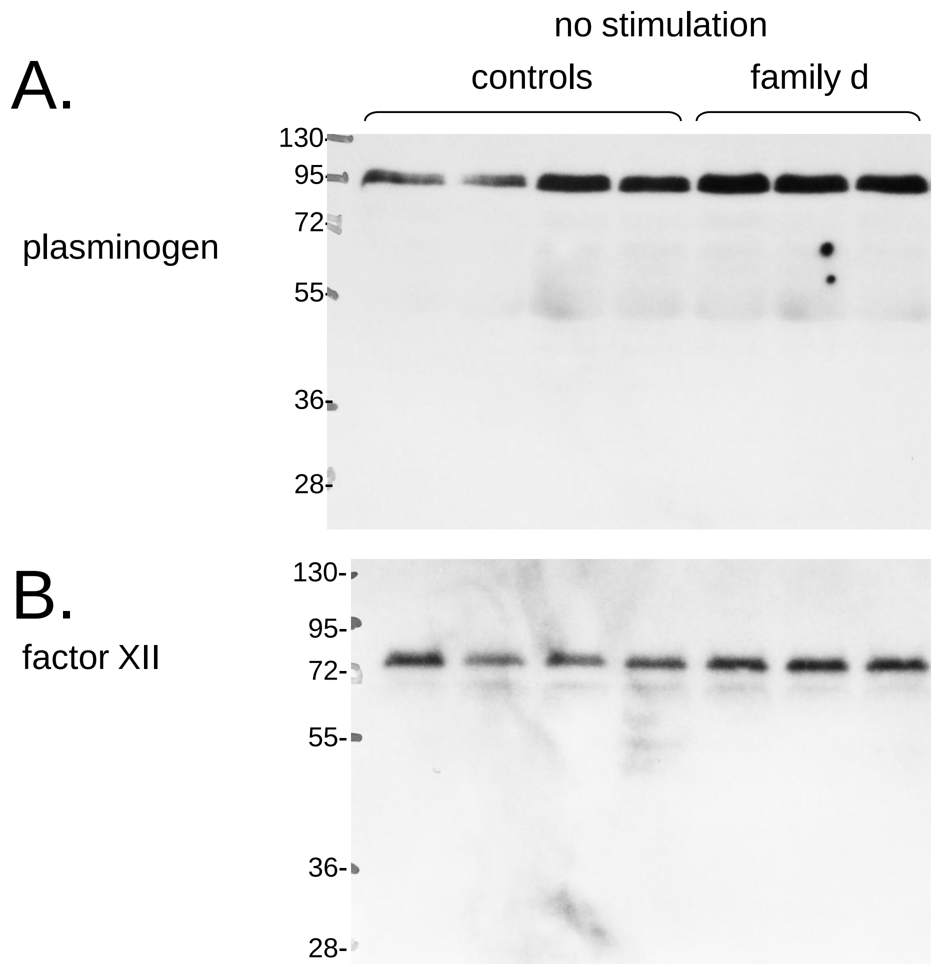

Supplement: Supplementary file 1 [file Data_Sheet_1.PDF]
